# Modified Sequential Deposition Route through Localized-Liquid-Liquid-Diffusion for Improved Perovskite Multi-Crystalline Thin Films with Micrometer-Scaled Grains for Solar Cells

**DOI:** 10.3390/nano8060416

**Published:** 2018-06-09

**Authors:** Tao Ling, Xiaoping Zou, Jin Cheng, Xiao Bai, Haiyan Ren, Dan Chen

**Affiliations:** Research Center for Sensor Technology, Beijing Key Laboratory for Sensor, Ministry of Education Key Laboratory for Modern Measurement and Control Technology, School of Applied Sciences, Beijing Information Science and Technology University, Jianxiangqiao Campus, Beijing 100101, China; 15652869292@163.com (T.L.); nanocheng@163.com (J.C.); baixiao_edu@163.com (X.B.); 18801139192@163.com (H.R.); 13436934850@139.com (D.C.);

**Keywords:** localized-liquid-liquid-diffusion, modified sequential deposition route, perovskite thin film, solar cells

## Abstract

High-class perovskite film with beautiful surface morphology (such as large-size grain, low defect density, good continuity and flatness) is normally believed to be a very important factor for high-efficiency perovskite solar cells (PSCs). Here, we report a modified sequential deposition route through localized-liquid-liquid-diffusion (LLLD) for qualified perovskite multi-crystalline thin films with micrometer-scaled grains for solar cells. We adopted a contact-type drop method to drop Methylammonium iodide (MAI) solution and have successfully used high-concentration MAI solution (73 mg/mL) to transform PbI_2_ film into high-class perovskite film via our route. A high efficiency of 10.7% was achieved for the device with spongy carbon film deposited on a separated FTO-substrate as a counter electrode under one sun illumination, which is the highest efficiency (as 2.5 times as previous efficiency) ever recorded in perovskite solar cells with a such spongy carbon/FTO composite counter electrode. The preparation techniques of high-class perovskite thin films under ambient conditions and the cheap spongy carbon/FTO composite counter electrode are beneficial for large-scale applications and commercialization.

## 1. Introduction

In recent years, perovskite solar cells (PSCs) have been recognized as the most promising alternative to conventional photovoltaic devices due to their high efficiency and simple process [1,2,3]. High-class perovskite thin film, with beautiful surface morphology (such as large-size grain, low defect density, good continuity, and flatness), is normally believed to be a very important factor for high-efficiency perovskite solar cells [4,5,6,7,8]. M. Grätzel et al. firstly developed the sequential deposition method (so-called two-step method) to prepare CH_3_NH_3_PbI_3_ perovskite thin films [9]. Since then, a lot of effort has been paid to developing the two-step method, similar to the two-step spin-coating process [10,11,12], and Xu et al. have demonstrated that the two-step spin coating method enables perovskite layer morphology to control and easily fabricate products [13]. Previous researchers prepared perovskite films via the conventional two-step coating method with a necessary step of PbI_2_ layer annealing [9,10,11,12,13]. In fact, we found the properties of the perovskite thin film are very sensitive to the annealing time of PbI_2_ layer especially under the dry ambient conditions used in our experiments.

Xu et al.’s previous study on the effect of humidity on the crystallization of two-step spin-coated perovskites suggested that appropriate moisture can facilitate the reaction between PbI_2_ and MAX (X = I, Cl), whereas the PbI_2_ film reacts with MAX slowly a under dry inert atmosphere [14]. H_2_O helps MAI to penetrate into the thick PbI_2_ to form thick film with a pure MAPbI_3_ phase and produce bigger gains by slowing down the perovskite crystallization rate [15]. However, the dry atmosphere (~10% relative humidity) was our experimental condition.

**Figure 1:**
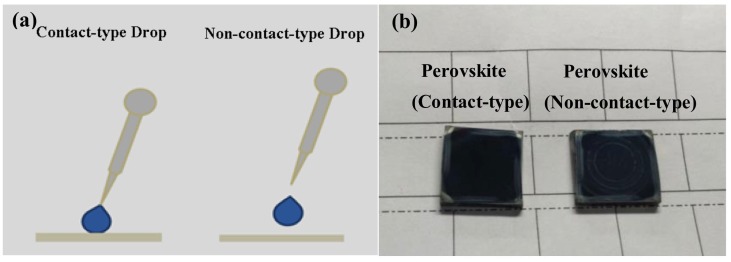
Schematic illustrations for drop method and picture of perovskite films: (**a**) Schematic illustrations for contact-type method and non-contact-type method to drop MAI solution; (**b**) Picture of perovskite films dropped by contact-type method and non-contact-type method.

In order to explore the effect of the annealing time of PbI_2_ thin film on properties of corresponding perovskite thin film, we annealed PbI_2_ film at 70 °C for 0 s, 10 s, and 20 s, respectively. We found that the 0 s annealing PbI_2_ film was more conducive to the formation of high-class perovskite films in our experiment, which means that the sequential deposition route through LLLD is more conducive to the formation of qualified perovskite multi-crystalline films. In addition, we adopted a contact-type drop method to drop MAI solution in order to reduce the damage of MAI solution to PbI_2_ thin liquid film (illustrated in Figure 1).

Previously, if MAI concentration is too high (≥70 mg/mL), most of the PbI_2_ precursor gets dissolved and exists as PbI_4_^2−^ in the solution, and only very little can reprecipitate to form MAPbI_3_ nanostructures [16]. Im et al. found that the size of the MAPbI_3_ cuboids increases with decreasing CH_3_NH_3_I concentration [17]. However, the MAI concentration in our experiment was 73 mg/mL and the corresponding MAI solution also successfully transformed PbI_2_ film into high-class perovskite film via LLLD. A high-class perovskite film with micrometer-scaled grain, low defect density, densification, and flatness was obtained by our process.

The tune of the formation process and composition of the light-absorber layer in PSCs has contributed to obtaining certified power conversion efficiencies (PCE) > 20% [18,19]. These PCEs have been obtained while perovskite solar cells were prepared using the single-step method (in combination with solvent engineering) or sequential deposition method or vapor deposition with an important step of device fabrication being the thermal evaporation of metals to form the counter electrode (CE). However, the cost of these metallic CEs is prohibitively high for large-scale applications and commercialization. 

Up to now, some researchers have developed carbon CEs to cut the costs of perovskite devices [20,21,22,23]. Yang et al. prepared a 2.6%-efficient perovskite solar cell with candle soot film deposited on a separated substrate as the carbon/FTO composite counter electrode [22]. In our research group, previously, a spongy carbon/FTO composite structure was adopted as a counter electrode and the corresponding cell achieved 4.24% power conversion efficiency (PCE) [23]. The perovskite solar cell was prepared via a modified sequential deposition route through LLLD under dry ambient conditions and got a 10.7% PCE in the size of 0.2 cm^2^, which is the highest efficiency ever recorded in perovskite solar cells with such a spongy carbon/FTO composite counter electrode.

## 2. Materials and Methods

### 2.1. Materials

Fluorine-doped SnO_2_ (FTO) substrates were obtained from Dalian HeptaChroma Solar Technology Development Corp (Dalian, China). *N*,*N*-dimethylformamide (DMF) and dimethyl sulfoxide (DMSO) were purchased from Sa’en Chemical Technology Corp (Shanghai, China). PbI_2_, 18NR-T TiO_2_ paste (mp-TiO_2_), acidic titanium dioxide solution (bl-TiO_2_), Methylammonium iodide (MAI), and isopropyl alcohol (IPA) were purchased from Shanghai MaterWin New Materials Corp, (Shanghai, China). Spiro-OMeTAD solution was purchased from Xi’an Polymer Light Technology Corp (Xi’an, China).

### 2.2. Device Fabrication

Fluorine-doped SnO_2_ (FTO) substrates were ultrasonically cleaned with mixed solution (detergent and deionized water), glass water (acetone:deionized water:2-propanol = 1:1:1), and alcohol, respectively. Before using, the FTO was cleaned by Ultraviolet Ozone (UVO) for 90 min. Compact TiO_2_ layer (bl-TiO_2_) was deposited on FTO substrate by spin-coating an acidic titanium dioxide solution at 2000 rpm for 60 s. Then the substrate was annealed on a hotplate at 100 °C for 10 min, and sintered in a muffle furnace at 500 °C for 30 min. Mesoporous TiO_2_ film (mp-TiO_2_) was coated on bl-TiO_2_ thin film by spin coating using a commercial 18NR-T TiO_2_ paste (4%, solid content) at 2000 rpm for 30 s. Subsequently, the substrate was dried on a hotplate at 100 °C for 10 min, and then sintered in a muffle furnace at 500 °C for 60 min. After that, 599.3 mg of PbI_2_ was dissolved in a component solvent (DMF:DMSO = 9.5:0.5) and then spin-coated onto a TiO_2_ (bl-TiO_2_ and mp-TiO_2_) layer at 1500 rpm for 30 s, and then annealed on a hotplate at 70 °C for 0 s, 10 s, and 20 s, respectively.

After the PbI_2_ film had cooled down to room temperature, the MAI solution (73 mg in 1 mL IPA) was spin coated onto the PbI_2_ layer at 1500 rpm for 30 s (without loading time), and then a thermal annealing of 150 °C for 15 min under low humidity air condition (~10% humidity) was processed. The hole transporting layer was deposited on top of the perovskite layer by 3000 rpm for 30 s using a commercial 2,2′,7,7′-tetrakis(*N*,*N*-dip-methoxyphenylamine)-9,9′-spirobifluorene (Spiro-OMeTAD) solution. Finally, some cleaned FTO glasses were used as substrates to obtain the soot of a burning candle as spongy carbon CEs. The spongy carbon films on FTO glasses were then pressed on the as-prepared uncompleted devices.

### 2.3. Characterization

An X-ray diffractometer (XRD) (Broker, D8 Focus, Beijing, China) was used to obtain XRD spectra from samples of perovskite films deposited on mp-TiO_2_/bl-TiO_2_/FTO substrates. The morphologies of these perovskite films were watched by a scanning electron microscope (SEM) (Zeiss SIGMA). The as-prepared devices were measured under full sun illumination (AM 1.5, 100 mW/cm^2^).

## 3. Results and Discussion

In order to reduce the possible damage of the MAI solution to PbI_2_ thin liquid film, we adopted a contact-type drop method to drop MAI solution (illustrated in Figure 1a), and the resulting perovskite films are shown in Figure 1b. In contrast to the non-contact-type drop method, this drop method reduced the damage of the MAI solution to PbI_2_ thin film according to Figure 1b.

**Figure 2:**
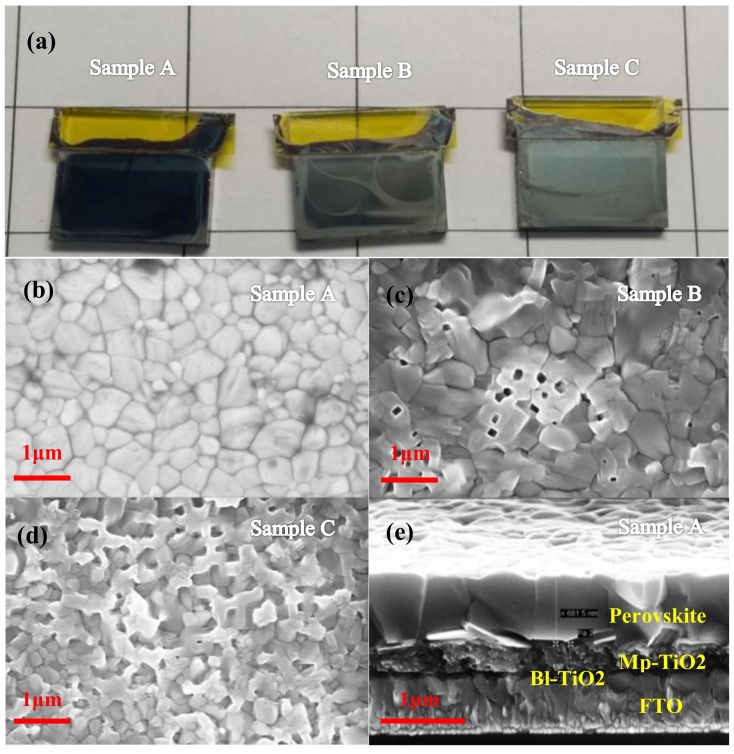
SEM images and picture: (**a**) Surface picture of perovskite films prepared by sequential deposition route with different PbI_2_ annealing times (0 s, 10 s and 20 s; corresponding sample A, sample B and sample C); (**b**–**d**) Surface SEM images of sample A, sample B and sample C; (**e**) Cross-sectional SEM image of sample A.

The perovskite films were prepared by sequential deposition route with PbI_2_ 0 s, 10 s, and 20 s annealing, which are named as sample A, sample B, and sample C, respectively. Scanning electron microscopy (SEM) images and image of perovskite layers prepared by two-step spin-coating with different PbI_2_ annealing times are shown in Figure 2. We found that the surfaces of the perovskite layers gradually became a white color with the increase of PbI_2_ annealing time, according to the image (Figure 2a) of the perovskite layers. From the surface SEM image (Figure 2b–d), it can be found that sample A showed obvious densification, sample B and C have double layers, and the bottom layer is dense and the top layer is loose. PbI_2_ solution film reacts with MAI solution to form a high-class perovskite thin film with beautiful surface morphology (such as large-size grain, low defect density, good continuity, and flatness) via LLLD, whereas, PbI_2_ solid film reacts with MAI solution to form loose perovskite thin film via localized-solid-liquid-diffusion (LSLD). The bottom layers of sample B and C are observed to contain large-size grain, but the top layer of sample B shows some small square holes and the top layer of sample C shows massive square unordered holes.

In the cross-sectional SEM image (Figure 2e) of sample A, we can see the FTO layer, bl-TiO_2_ layer, mp-TiO_2_ layer and perovskite layer clearly from the bottom to the top, and the longitudinal size of perovskite grains equal to the film thickness. This qualified perovskite film (sample A) indicates that the modified sequential deposition route through LLLD is beneficial to the growth of perovskite crystals under dry ambient condition and enhancement of the corresponding photovoltaic performance.

**Figure 3:**
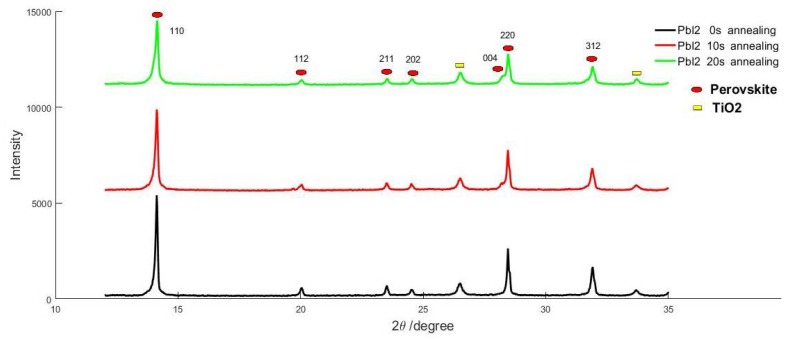
X-ray diffraction (XRD) patterns of perovskite films prepared by sequential deposition route with different PbI_2_ annealing times.

Figure 3 shows XRD pattern of the samples prepared by two-step spin-coating method with different PbI_2_ annealing times. According to the Figure 3, there are no peaks of PbI_2_ (corresponding to ~26°) and MAI (corresponding to ~19°), which means there are no residuals of PbI_2_ and MAI in the samples, and the compositions of perovskites in the samples are very pure. As the PbI_2_ annealing time increased to 10 s, the (004) peak of the tetragonal structure for CH_3_NH_3_PbI_3_ emerged, which indicates a phase transition from cubic to tetragonal structure [24]. This result suggest that the perovskite in sample A and bottom layers of sample B and C is cubic structure, and the perovskite in top layers of sample B and C is tetragonal structure. In addition, the intensity ratio (~2.06) of the highest peak (110) of sample A to its secondary peak (220) is lightly above that of others (~1.93 and ~2.01). The above points means the crystal structure of sample A is better than others and sequential deposition route through LLLD is more propitious to the formation of qualified perovskite multi-crystalline films, which can enhance the corresponding device efficiency. 

**Figure 4:**
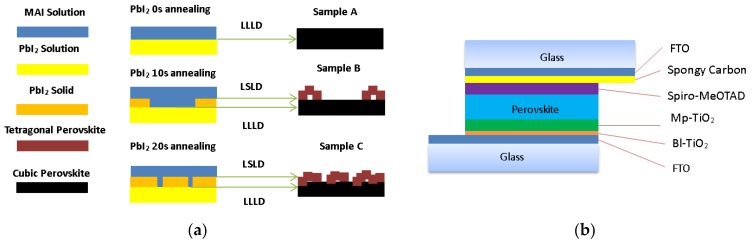
Schematic illustration for diffusion mechanisms and structure of devices: (**a**) Schematic illustration for the mechanisms of LLLD and LSLD for perovskite multi-crystalline film; (**b**) Structure of devices with low-cost spongy carbon/FTO composite counter electrode.

Schematic illustrations for LLLD and LSLD for qualified perovskite multi-crystalline film are shown in Figure 4a. As PbI_2_ annealing time increases, its solid part and the ratio of LLLD with MAI solution decreases. The changes in the morphology of the perovskite have finally happened. Due to the 0 s annealing PbI_2_ film was the solution state, and MAI could more easily diffuse to the PbI_2_ thin film interior via LLLD and transform PbI_2_ film into a high-class cubic structure perovskite film. Moreover, the PbI_2_ thin film had some of the solvent of DMF, which could facilitate perovskite conversion, improve the film morphology, and reduce crystal defects, and thus enhance charge transfer efficiency [25]. Due to the annealing time being relatively short, there are double layers in the 10 s annealing PbI_2_ film and 20 s annealing PbI_2_ film. The top layer is solid state, as a result of contacting with air during the spin-coating. MAI is difficult to diffuse to the solid top layer of the PbI_2_ thin film interior via LSLD and transform into poor perovskite film with a tetragonal structure. The bottom layer is in solution state for having not made contact with air during the spin-coating, which is similar to sample A, and can be transformed into a high-class cubic structure perovskite film. The hydrolysis of perovskite must be reduced because it was prepared in dry air. A combination of the above factors lead to the high-class perovskite films being fabricated by the sequential deposition route through LLLD under dry ambient conditions and the structure of the corresponding devices with the low-cost spongy carbon/FTO composite counter electrode is shown in Figure 4b.

**Figure 5:**
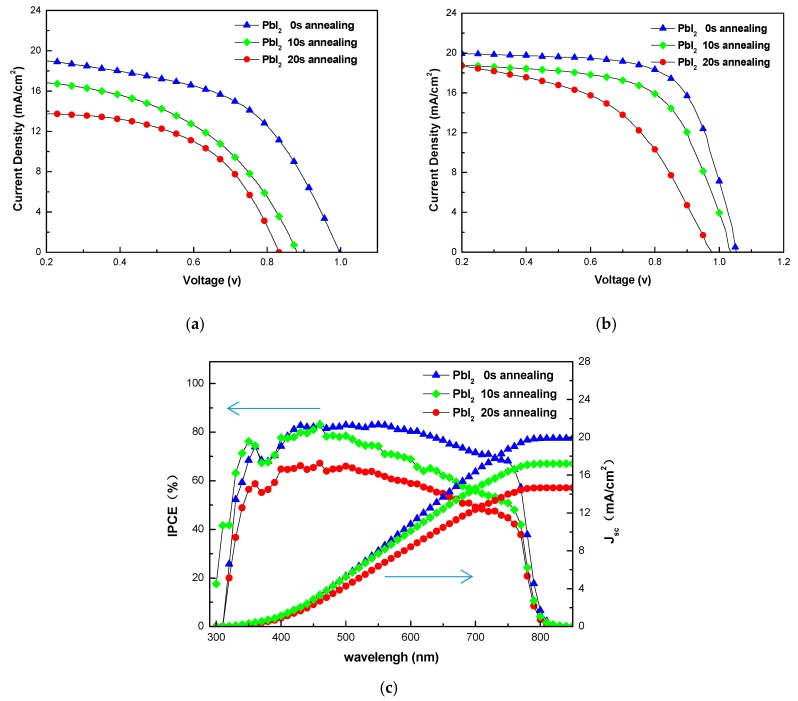
J-V curves from reverse scan (RS) and monochromatic incident photon-to-electron conversion efficiency (IPCE) of PSCs prepared by sequential deposition route with different PbI_2_ annealing times: (**a**) J-V curves for the devices with carbon/FTO composite electrode; (**b**) J-V curves for the devices with gold electrode; (**c**) IPCE of devices with carbon/FTO composite electrode.

Figure 5a,b show the light current-voltage (J-V) curves from reverse scan (RS) for the devices with a carbon/FTO composite electrode and gold electrode, measured under simulated sunlight with an intensity of 100 mW/cm^2^ (AM 1.5 G). According to the J-V curve, it is found that the device (corresponding to PbI_2_ 0 s annealing) via the modified sequential deposition route through LLLD showed the best performance in major parameters (such as V_oc_, J_sc_ and PCE, and their detailed values can be seen in Table 1) and the others showed obvious attenuation in major parameters. To be sure of the efficacy of our new coating processes and the reliability of current density data, we measured the IPCE of the devices with a carbon/FTO composite electrode, and the results are shown in Figure 5c. Generally, the device performance is increased with the decrease of PbI_2_ annealing times, indicating that the sequential deposition route through LLLD is more conducive to the formation of qualified perovskite multi-crystalline films under dry ambient conditions.

According to the data of Table 1, it can be found that the photovoltaic performance of contact-type processed PSCs is better than the non-contact-type processed PSCs. This further validates the reduced damage of the MAI solution to the PbI_2_ thin film when dropping the MAI solution via contact-type method. A high efficiency of 10.7% was achieved for the device with the carbon/FTO composite electrode. Although this efficiency is not the highest in PSCs, it is the highest efficiency (as 2.5 times as previous efficiency) ever recorded in PSCs with such a spongy carbon/FTO composite counter electrode. Furthermore, the preparation techniques of high-class perovskite thin films and the cheap spongy carbon/FTO composite counter electrode, under absolute ambient conditions, are beneficial for large-scale applications and commercialization.

## 4. Conclusions

A modified sequential deposition route through LLLD was more conducive to the formation of qualified perovskite multi-crystalline thin films with micrometer-scaled grain, low defect density, densification, and flatness under dry ambient condition. The two diffusion mechanisms of LLLD and LSLD were suggested and preliminarily confirmed. A contact-type drop method to drop MAI solution can reduce the damage of MAI solution to PbI_2_ thin films, and high-concentration MAI solution can also transform PbI_2_ film into high-class perovskite film via our route. Moreover, a perovskite solar cell was prepared via the modified sequential deposition route through LLLD under absolute ambient conditions and got a 10.7% PCE in a size of 0.2 cm^2^, which is the highest efficiency ever recorded in perovskite solar cells with such a spongy carbon/FTO composite counter electrode. Obviously, a great deal of production of high-efficiency perovskite solar cells, with a low-cost spongy carbon/FTO composite counter electrode, under an absolutely ambient atmosphere, is possible.

## Figures and Tables

**Table 1:** Parameters of contact-type or non-contact-type processed PSCs with carbon/FTO composite electrode or gold electrode prepared by sequential deposition route with different PbI_2_ annealing times under RS.

T ^a^ (s)	DM ^b^	EM ^c^	J_sc_^d^ (mA/cm^−2^)	V_oc_^e^ (v)	FF ^f^	PCE ^g^ (%)
0	non-contact-type	carbon/FTO	17.19	0.95	0.48	7.89
	contact-type	carbon/FTO	19.89	1.00	0.54	10.70
	non-contact-type	gold	19.69	1.02	0.66	13.29
	contact-type	gold	20.18	1.05	0.70	14.86
10	contact-type	carbon/FTO	17.49	0.89	0.49	7.64
	contact-type	gold	19.11	1.04	0.64	12.73
20	contact-type	carbon/FTO	14.22	0.84	0.56	6.66
	contact-type	gold	19.66	0.98	0.51	9.73

^a^ T: PbI_2_ annealing times; ^b^ DM: Drop method of MAI solution; ^c^ EM: Electrode materials; ^d^ J_sc_: Short-circuit photocurrent density; ^e^ V_oc_: Open-circuit voltage; ^f^ FF: Fill factor; ^g^ PCE: Power conversion efficiency.

## References

[1] Park N.-G. (2013). Organometal perovskite light absorbers toward a 20% efficiency low-cost solid-state mesoscopic solar cell. J. Phys. Chem. Lett..

[2] Yin X., Guo Y.J., Xue Z., Xu P., He M., Liu B. (2015). Performance enhancement of perovskite-sensitized mesoscopic solar cells using Nb-doped TiO_2_ compact layer. Nano Res..

[3] Yang W.S., Noh J.H., Jeon J.N., Kim Y.C., Ryu S.C., Seo S.J., Seok S.I.I. (2015). High-performance photovoltaic perovskite layers fabricated through intramolecular exchange. Science.

[4] Stranks S.D., Eperon G.E., Grancini G., Menelaou C., Alcocer M.J.P., Leijtens T., Herz L.M., Petrozza A., Snaith H.J. (2013). Electron-Hole Diffusion Lengths Exceeding 1 Micrometer in an Organometal Trihalide Perovskite Absorber. Science.

[5] Xing G., Mathews N., Sun S., Lim S.S., Lam Y.M., Gratzel M., Mhaisalkar S., Sum T.C. (2013). Long-range Balanced Electron- and Hole-Transport Lengths in Organic-Inorganic CH_3_NH_3_PbI_3_. Science.

[6] Eperon G.E., Burlakov V.M., Docampo P., Goriely A., Snaith H.J. (2014). Morphological Control for High Performance, Solution-Processed Planar Heterojunction Perovskite Solar Cells. Adv. Funct. Mater..

[7] Chen Z., Li H., Tang Y., Huang X., Ho D., Lee C.-S. (2014). Shape-Controlled Synthesis of Organolead Halide Perovskite Nanocrystals and Their Tunable Optical Absorption. Mater. Res. Express.

[8] Yin W.J., Shi T., Yan Y. (2014). Unique Properties of Halide Perovskites as Possible Origins of the Superior Solar Cell Performance. Adv. Mater..

[9] Burschka J., Pellet N., Moon S.-J., Humphry-Baker R., Gao P., Nazeeruddin M.K., Grätzel M. (2013). Sequential Deposition as a Route to High-Performance Perovskite-Sensitized Solar Cells. Nature.

[10] Xiao Z., Dong Q., Bi C., Shao Y., Yuan Y., Huang J. (2014). Solvent Annealing of Perovskite-Induced Crystal Growth for Photovoltaic-Device Efficiency Enhancement. Adv. Mater..

[11] Xiao Z., Bi C., Shao Y., Dong Q., Wang Q., Yuan Y., Wang C., Gao Y., Huang J. (2014). Efficient, High Yield Perovskite Photovoltaic Devices Grown by Interdiffusion of Solution-Processed Precursor Stacking Layers. Energy Environ. Sci..

[12] Chae J., Dong Q., Huang J., Centrone A. (2015). Chloride Incorporation Process in CH_3_NH_3_PbI_3-x_Cl_x_ Perovskites via Nanoscale Bandgap Maps. Nano Lett..

[13] Xu Y., Zhu L., Shi J., Lv S., Xu X., Xiao J., Dong J., Wu H., Luo Y., Li D., Meng Q. (2015). Efficient Hybrid Mesoscopic Solar Cells with Morphology-Controlled CH_3_NH_3_PbI_3-x_Cl_x_ Derived from Two-Step Spin Coating Method. ACS Appl. Mater. Interfaces.

[14] Xu Y., Zhu L., Shi J., Xu X., Xiao J., Dong J., Wu H., Luo Y., Li D., Meng Q. (2016). The Effect of Humidity upon the Crystallization Process of Two-Step Spin-Coated Organic-Inorganic Perovskites. ChemPhysChem.

[15] Chiang C.H., Nazeeruddin M.K., Grätzel M., Wu C.G. (2017). The synergistic effect of H_2_O and DMF towards stable and 20% efficiency inverted perovskite solar cells. Energy Environ. Sci..

[16] Fu Y., Meng F., Rowley M.B., Thompson B.J., Shearer M.J., Ma D., Hamers R.J., Wright J.C., Jin S. (2015). Solution growth of single crystal methylammonium lead halide perovskite nanostructures for optoelectronic and photovoltaic applications. J. Am. Chem. Soc..

[17] Im J.H., Jang I.H., Pellet N., Grätzel M., Park N.G. (2014). Growth of CH_3_NH_3_PbI_3_ cuboids with controlled size for high-efficiency perovskite solar cells. Nat. Nanotechnol..

[18] Kojima A., Teshima K., Shirai Y., Miyasaka T. (2009). Organometal halide perovskites as visible-light sensitizers for photovoltaic cells. J. Am. Chem. Soc..

[19] Yang W.S., Park B.-W., Jung E.H., Jeon N.J., Kim Y.C., Lee D.U., Shin S.S., Seo J., Kim E.K., Noh J.H. (2017). Iodide management in formamidinium-lead-halide-based perovskite layers for efficient solar cells. Science.

[20] Mei A., Li X., Liu L., Ku Z., Liu T., Rong Y.G., Xu M., Hu M., Chen J., Yang Y. (2014). A hole-conductor-free, fully printable mesoscopic perovskite solar cell with high stability. Science.

[21] Habisreutinger S.N., Leijtens T., Eperon G.E., Stranks S.D., Nicholas R.J., Snaith H.J. (2014). Carbon nanotube/polymer composites as a highly stable hole collection layer in perovskite solar cells. Nano Lett..

[22] Wei Z., Yan K., Chen H., Yi Y., Zhang T., Long X., Li J., Zhang L., Wang J., Yang S. (2014). Cost-efficient clamping solar cells using candle soot for hole extraction from ambipolar perovskites. Energy Environ. Sci..

[23] Zhang N., Guo Y., Yin X., He M., Zou X. (2016). Spongy carbon film deposited on a separated substrate as counter electrode for perovskite-based solar cell. Mater. Lett..

[24] Luo D., Yu L., Wang H., Zou T., Luo L., Liu Z., Lu Z. (2015). Cubic structure of the mixed halide perovskite CH_3_NH_3_PbI_3-x_Cl_x_ via thermal annealing. RSC Adv..

[25] Wu J., Xu X., Zhao Y., Shi J., Xu Y., Luo Y., Li D., Wu H., Meng Q. (2017). DMF as an additive in two-step spin coating method for 20% conversion efficiency perovskite solar cells. ACS Appl. Mater. Interfaces.

